# Balsam of Peru in Pruritis

**Published:** 1880-03

**Authors:** 


### BALSAM OF PERU IN PRURITIS.

Dr. Auerbach has treated pruritis with this substance with the greatest success. It is well rubbed into the parts, and cure results in a few days.—*Deutsche Med. Woche.*

